# *N*′-[(*E*)-1-(5-Chloro-2-hy­droxy­phen­yl)ethyl­idene]pyridine-3-carbohydrazide monohydrate

**DOI:** 10.1107/S1600536810025213

**Published:** 2010-06-30

**Authors:** Abid Hussain, Zahid Shafiq, M. Nawaz Tahir, Muhammad Yaqub

**Affiliations:** a Department of Chemistry, Bahauddin Zakariya University, Multan 60800, Pakistan; b Department of Physics, University of Sargodha, Sargodha, Pakistan

## Abstract

In the title compound, C_14_H_12_ClN_3_O_2_·H_2_O, the benzene ring and the pyridine rings are oriented at a dihedral angle of 57.73 (12)° and an intra­molecular O—H⋯N hydrogen bond generates an *S*(6) ring. In the crystal, the water mol­ecule forms O—H⋯O and O—H⋯N hydrogen bonds to the organic mol­ecule, leading to chains containing *R*_4_^4^(16) loops. In addition, weak aromatic π–π stacking inter­actions between the centroids of pyridine rings [at distance of 3.864 (2) and 4.013 (2) Å] and C—H⋯π inter­actions occur.

## Related literature

For background to Schiff bases and for related structures, see: Shafiq *et al.* (2009*a* ▶,*b* ▶): For graph-set notation, see: Bernstein *et al.* (1995 ▶).
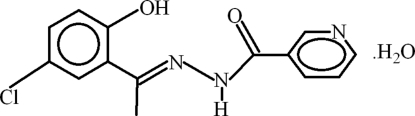


## Experimental

### 

#### Crystal data

C_14_H_12_ClN_3_O_2_·H_2_O
*M* *_r_* = 307.73
Triclinic, 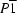

*a* = 7.1693 (5) Å
*b* = 7.4964 (4) Å
*c* = 14.5966 (9) Å
α = 90.138 (2)°
β = 95.835 (1)°
γ = 115.755 (2)°
*V* = 701.94 (8) Å^3^
*Z* = 2
Mo *K*α radiation
μ = 0.29 mm^−1^
*T* = 296 K
0.28 × 0.18 × 0.14 mm

#### Data collection

Bruker Kappa APEXII CCD diffractometer
Absorption correction: multi-scan (*SADABS*; Bruker, 2005 ▶) *T*_min_ = 0.942, *T*_max_ = 0.959
10105 measured reflections
2491 independent reflections
2151 reflections with *I* > 2σ(*I*)
*R*_int_ = 0.027

#### Refinement

*R*[*F* ^2^ > 2σ(*F* ^2^)] = 0.047
*wR*(*F* ^2^) = 0.135
*S* = 1.17
2491 reflections
198 parameters
H atoms treated by a mixture of independent and constrained refinement
Δρ_max_ = 0.24 e Å^−3^
Δρ_min_ = −0.35 e Å^−3^

### 

Data collection: *APEX2* (Bruker, 2009 ▶); cell refinement: *SAINT* (Bruker, 2009 ▶); data reduction: *SAINT*; program(s) used to solve structure: *SHELXS97* (Sheldrick, 2008 ▶); program(s) used to refine structure: *SHELXL97* (Sheldrick, 2008 ▶); molecular graphics: *ORTEP-3* (Farrugia, 1997 ▶) and *PLATON* (Spek, 2009 ▶); software used to prepare material for publication: *WinGX* (Farrugia, 1999 ▶) and *PLATON*.

## Supplementary Material

Crystal structure: contains datablocks global, I. DOI: 10.1107/S1600536810025213/hb5528sup1.cif

Structure factors: contains datablocks I. DOI: 10.1107/S1600536810025213/hb5528Isup2.hkl

Additional supplementary materials:  crystallographic information; 3D view; checkCIF report

## Figures and Tables

**Table 1:** Hydrogen-bond geometry (Å, °) *Cg*2 is the centroid of the C1–C6 phenyl ring.

*D*—H⋯*A*	*D*—H	H⋯*A*	*D*⋯*A*	*D*—H⋯*A*
O1—H1⋯N1	0.82	1.84	2.555 (3)	144
N2—H2⋯O3^i^	0.86	2.06	2.898 (4)	166
O3—H3*A*⋯O2	0.89 (5)	1.88 (5)	2.760 (4)	171 (3)
O3—H3*B*⋯N3^ii^	0.91 (4)	2.01 (4)	2.885 (4)	161 (4)
C8—H8*A*⋯*Cg*2^iii^	0.96	2.99	3.763 (4)	139

Symmetry codes: (i) 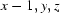; (ii) 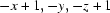; (iii) 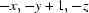.

**Fig. 1.**
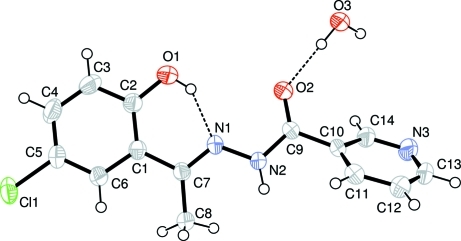
View of the title compound with displacement ellipsoids drawn at the 50% probability level. The dotted line shows the intramolecular H-bond.

**Fig. 2.**
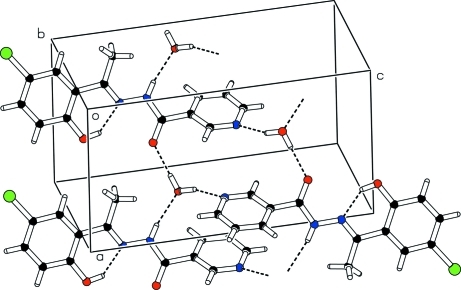
The partial packing of (I), which shows that molecules form infinite one dimensional polymeric chains with different ring motifs.

## supplementary crystallographic information

### Comment

We have reported crystal structures of Schiff bases containing pyridne (Shafiq *et al.*, 2009*a*, 2009*b*) and as a part of this project, we report herein the structure and synthesis of the title compound (I, Fig. 1).

In (I) the group A (C1–C8/O1/CL1) of 5-chloro-2-hydroxyacetophenone, the central group B (N1/N2/C9/O2) and the pyridine ring C (C10—C14/N3) are planar with r. m. s. deviation of 0.0330, 0.0182 and 0.0082 Å, respectively. The dihedral angle between A/B, A/C and B/C is 6.62 (11), 58.08 (10) and 52.98 (14)°, respectively. There exist intramolecular H-bonding of O—H···N type forming an S(6) ring motif (Bernstein *et al.*, 1995). The water molecule acts as donar as well as accepter and therefore interconnects three molecules. Due to intra as well as intermolecular H-bondings of O—H···O and O—H···N types (Table 1, Fig. 2), the title compound is stabilized in infinite one dimensional polymeric chains. In the polymeric chains *R*_4_^4^(16) ring motifs are formed. The π–π interactions exist between the centroids of pyridine rings at distance of 3.864 (2) Å [symmetry: -*x*, -*y*, 1 - *z*] and at 4.013 (2) Å [symmetry: 1 - *x*, 1 - *y*, 1 - *z*]. The C—H···π interaction (Table 1) also plays an important role in stabilizing the structure.

### Experimental

To a hot stirred solution of 5-chloro-2-hydroxyacetophenone (1.71 g, 0.01 mole) in ethanol, 25 ml nicotinic acid hydrazide (1.37 g, 0.01 mol) was added. The resultant mixture was then heated under reflux for 7–8 h. The reaction was monitored through TLC. The precipitate were formed were collected by suction filteration. The resultant crude material was dried and recrystalized in 1,4-dioxan:ethanol(1:2) to affoard light brown needles of (I).

### Refinement

The coordinates of H-atoms of water molecule were refined. The H-atoms were positioned geometrically (O–H = 0.82, N–H = 0.86, C–H = 0.93–0.96 Å) and refined as riding with *U*_iso_(H) = *xU*_eq_(C, N, O), where *x* = 1.5 for methyl and *x* = 1.2 for all other H-atoms.

### Crystal data

**Table:**

C_14_H_12_ClN_3_O_2_·H_2_O	*Z* = 2
*M**_r_* = 307.73	*F*(000) = 320
Triclinic, *P*1	*D*_x_ = 1.456 Mg m^−^^3^
Hall symbol: -P 1	Mo *K*α radiation, λ = 0.71073 Å
*a* = 7.1693 (5) Å	Cell parameters from 1770 reflections
*b* = 7.4964 (4) Å	θ = 2.6–28.4°
*c* = 14.5966 (9) Å	µ = 0.29 mm^−^^1^
α = 90.138 (2)°	*T* = 296 K
β = 95.835 (1)°	Needle, light brown
γ = 115.755 (2)°	0.28 × 0.18 × 0.14 mm
*V* = 701.94 (8) Å^3^	

### Data collection

**Table:**

Bruker Kappa APEXII CCD diffractometer	2491 independent reflections
Radiation source: fine-focus sealed tube	2151 reflections with *I* > 2σ(*I*)
graphite	*R*_int_ = 0.027
Detector resolution: 8.20 pixels mm^-1^	θ_max_ = 25.3°, θ_min_ = 2.8°
ω scans	*h* = −8→8
Absorption correction: multi-scan (*SADABS*; Bruker, 2005)	*k* = −8→8
*T*_min_ = 0.942, *T*_max_ = 0.959	*l* = −16→17
10105 measured reflections	

### Refinement

**Table:**

Refinement on *F*^2^	Primary atom site location: structure-invariant direct methods
Least-squares matrix: full	Secondary atom site location: difference Fourier map
*R*[*F*^2^ > 2σ(*F*^2^)] = 0.047	Hydrogen site location: inferred from neighbouring sites
*wR*(*F*^2^) = 0.135	H atoms treated by a mixture of independent and constrained refinement
*S* = 1.17	*w* = 1/[σ^2^(*F*_o_^2^) + (0.0375*P*)^2^ + 0.8617*P*] where *P* = (*F*_o_^2^ + 2*F*_c_^2^)/3
2491 reflections	(Δ/σ)_max_ < 0.001
198 parameters	Δρ_max_ = 0.24 e Å^−^^3^
0 restraints	Δρ_min_ = −0.35 e Å^−^^3^

### Special details

**Table:**

Geometry. Bond distances, angles *etc*. have been calculated using the rounded fractional coordinates. All su's are estimated from the variances of the (full) variance-covariance matrix. The cell e.s.d.'s are taken into account in the estimation of distances, angles and torsion angles
Refinement. Refinement of *F*^2^ against ALL reflections. The weighted *R*-factor *wR* and goodness of fit *S* are based on *F*^2^, conventional *R*-factors *R* are based on *F*, with *F* set to zero for negative *F*^2^. The threshold expression of *F*^2^ > σ(*F*^2^) is used only for calculating *R*-factors(gt) *etc*. and is not relevant to the choice of reflections for refinement. *R*-factors based on *F*^2^ are statistically about twice as large as those based on *F*, and *R*- factors based on ALL data will be even larger.

### Fractional atomic coordinates and isotropic or equivalent isotropic displacement parameters (Å^2^)

**Table:**

	*x*	*y*	*z*	*U*_iso_*/*U*_eq_	
Cl1	−0.33027 (14)	0.26260 (13)	−0.24936 (5)	0.0523 (3)	
O1	0.3445 (3)	0.2717 (4)	0.01706 (15)	0.0491 (8)	
O2	0.4191 (3)	0.2364 (4)	0.25756 (15)	0.0544 (8)	
N1	0.1131 (3)	0.2425 (3)	0.14368 (15)	0.0338 (7)	
N2	0.0986 (4)	0.2273 (4)	0.23701 (15)	0.0347 (7)	
N3	0.2974 (4)	0.0892 (5)	0.53373 (18)	0.0523 (10)	
C1	−0.0064 (4)	0.2462 (4)	−0.01053 (18)	0.0311 (8)	
C2	0.1854 (4)	0.2739 (4)	−0.04006 (19)	0.0365 (9)	
C3	0.2156 (5)	0.3040 (5)	−0.1323 (2)	0.0505 (11)	
C4	0.0595 (6)	0.3026 (5)	−0.1958 (2)	0.0502 (11)	
C5	−0.1289 (5)	0.2703 (4)	−0.16794 (19)	0.0391 (9)	
C6	−0.1630 (4)	0.2451 (4)	−0.07681 (19)	0.0347 (8)	
C7	−0.0461 (4)	0.2199 (4)	0.08716 (18)	0.0306 (8)	
C8	−0.2576 (5)	0.1709 (6)	0.1141 (2)	0.0476 (10)	
C9	0.2671 (4)	0.2336 (4)	0.28988 (19)	0.0343 (9)	
C10	0.2551 (4)	0.2337 (4)	0.39173 (18)	0.0341 (9)	
C11	0.2132 (4)	0.3709 (5)	0.4384 (2)	0.0398 (9)	
C12	0.2188 (5)	0.3679 (5)	0.5328 (2)	0.0500 (11)	
C13	0.2599 (5)	0.2246 (6)	0.5769 (2)	0.0537 (13)	
C14	0.2966 (5)	0.0971 (5)	0.4425 (2)	0.0421 (10)	
O3	0.7770 (3)	0.2277 (4)	0.34221 (16)	0.0478 (8)	
H1	0.31433	0.26059	0.07012	0.0736*	
H2	−0.01187	0.21446	0.26025	0.0416*	
H3	0.34320	0.32529	−0.15140	0.0608*	
H4	0.08174	0.32343	−0.25733	0.0605*	
H6	−0.29067	0.22721	−0.05904	0.0416*	
H8A	−0.27145	0.29111	0.12362	0.0715*	
H8B	−0.27573	0.10118	0.17002	0.0715*	
H8C	−0.36158	0.08889	0.06588	0.0715*	
H11	0.18180	0.46339	0.40644	0.0478*	
H12	0.19538	0.46062	0.56616	0.0598*	
H13	0.26161	0.22236	0.64064	0.0645*	
H14	0.32550	0.00545	0.41125	0.0505*	
H3A	0.657 (6)	0.217 (5)	0.314 (3)	0.0574*	
H3B	0.735 (6)	0.139 (5)	0.387 (3)	0.0574*	

### Atomic displacement parameters (Å^2^)

**Table:**

	*U*^11^	*U*^22^	*U*^33^	*U*^12^	*U*^13^	*U*^23^
Cl1	0.0600 (5)	0.0632 (5)	0.0309 (4)	0.0269 (4)	−0.0066 (3)	0.0055 (3)
O1	0.0391 (12)	0.0743 (16)	0.0436 (12)	0.0329 (12)	0.0093 (10)	0.0112 (12)
O2	0.0426 (12)	0.1011 (19)	0.0355 (12)	0.0456 (13)	0.0069 (10)	0.0129 (12)
N1	0.0346 (12)	0.0435 (14)	0.0252 (12)	0.0192 (11)	0.0011 (9)	0.0024 (10)
N2	0.0313 (12)	0.0509 (14)	0.0248 (12)	0.0209 (11)	0.0026 (9)	0.0032 (10)
N3	0.0568 (17)	0.079 (2)	0.0347 (14)	0.0416 (16)	0.0083 (12)	0.0156 (14)
C1	0.0344 (14)	0.0286 (14)	0.0287 (14)	0.0127 (12)	0.0015 (11)	0.0001 (11)
C2	0.0393 (16)	0.0394 (16)	0.0347 (15)	0.0204 (13)	0.0056 (12)	0.0032 (12)
C3	0.0509 (19)	0.069 (2)	0.0438 (18)	0.0342 (18)	0.0205 (15)	0.0110 (16)
C4	0.067 (2)	0.063 (2)	0.0297 (16)	0.0352 (19)	0.0135 (15)	0.0081 (14)
C5	0.0493 (18)	0.0391 (16)	0.0281 (14)	0.0199 (14)	−0.0012 (12)	0.0013 (12)
C6	0.0338 (14)	0.0366 (15)	0.0316 (15)	0.0141 (12)	0.0012 (11)	0.0001 (12)
C7	0.0314 (14)	0.0326 (14)	0.0282 (14)	0.0148 (12)	0.0015 (11)	0.0004 (11)
C8	0.0356 (16)	0.079 (2)	0.0296 (15)	0.0264 (16)	0.0031 (12)	0.0055 (15)
C9	0.0307 (14)	0.0450 (17)	0.0301 (14)	0.0197 (13)	0.0014 (11)	0.0039 (12)
C10	0.0270 (13)	0.0469 (17)	0.0267 (14)	0.0153 (12)	−0.0004 (11)	0.0020 (12)
C11	0.0356 (15)	0.0473 (17)	0.0368 (16)	0.0192 (14)	0.0000 (12)	0.0014 (13)
C12	0.0470 (18)	0.068 (2)	0.0382 (17)	0.0284 (17)	0.0032 (14)	−0.0074 (16)
C13	0.053 (2)	0.092 (3)	0.0261 (16)	0.041 (2)	0.0038 (14)	0.0042 (16)
C14	0.0458 (17)	0.057 (2)	0.0322 (16)	0.0302 (16)	0.0057 (13)	0.0064 (14)
O3	0.0364 (12)	0.0757 (17)	0.0380 (12)	0.0304 (12)	0.0052 (9)	0.0118 (11)

### Geometric parameters (Å, °)

**Table:**

Cl1—C5	1.755 (4)	C5—C6	1.376 (4)
O1—C2	1.349 (4)	C7—C8	1.492 (5)
O2—C9	1.223 (4)	C9—C10	1.498 (4)
O1—H1	0.8200	C10—C11	1.385 (4)
O3—H3A	0.89 (5)	C10—C14	1.382 (4)
O3—H3B	0.91 (4)	C11—C12	1.375 (4)
N1—N2	1.378 (3)	C12—C13	1.377 (5)
N1—C7	1.285 (4)	C3—H3	0.9300
N2—C9	1.348 (4)	C4—H4	0.9300
N3—C14	1.333 (4)	C6—H6	0.9300
N3—C13	1.330 (5)	C8—H8C	0.9600
N2—H2	0.8600	C8—H8A	0.9600
C1—C7	1.479 (4)	C8—H8B	0.9600
C1—C6	1.403 (4)	C11—H11	0.9300
C1—C2	1.412 (4)	C12—H12	0.9300
C2—C3	1.389 (4)	C13—H13	0.9300
C3—C4	1.375 (5)	C14—H14	0.9300
C4—C5	1.370 (6)		
			
C2—O1—H1	109.00	C9—C10—C14	119.0 (3)
H3A—O3—H3B	102 (4)	C9—C10—C11	122.9 (3)
N2—N1—C7	120.6 (3)	C10—C11—C12	118.8 (3)
N1—N2—C9	116.3 (3)	C11—C12—C13	118.5 (3)
C13—N3—C14	116.9 (3)	N3—C13—C12	123.9 (3)
N1—N2—H2	122.00	N3—C14—C10	123.7 (3)
C9—N2—H2	122.00	C2—C3—H3	120.00
C2—C1—C6	118.2 (2)	C4—C3—H3	120.00
C2—C1—C7	122.3 (3)	C5—C4—H4	120.00
C6—C1—C7	119.5 (3)	C3—C4—H4	120.00
C1—C2—C3	119.6 (3)	C1—C6—H6	120.00
O1—C2—C3	117.1 (3)	C5—C6—H6	120.00
O1—C2—C1	123.3 (3)	C7—C8—H8B	109.00
C2—C3—C4	120.9 (3)	C7—C8—H8C	109.00
C3—C4—C5	119.7 (3)	H8A—C8—H8B	110.00
Cl1—C5—C6	119.2 (3)	H8A—C8—H8C	109.00
Cl1—C5—C4	119.7 (2)	H8B—C8—H8C	109.00
C4—C5—C6	121.1 (3)	C7—C8—H8A	109.00
C1—C6—C5	120.4 (3)	C10—C11—H11	121.00
N1—C7—C8	124.7 (2)	C12—C11—H11	121.00
C1—C7—C8	120.6 (3)	C13—C12—H12	121.00
N1—C7—C1	114.7 (3)	C11—C12—H12	121.00
O2—C9—N2	122.8 (3)	N3—C13—H13	118.00
O2—C9—C10	121.7 (3)	C12—C13—H13	118.00
N2—C9—C10	115.5 (3)	N3—C14—H14	118.00
C11—C10—C14	118.1 (3)	C10—C14—H14	118.00
			
C7—N1—N2—C9	173.6 (3)	O1—C2—C3—C4	178.1 (3)
N2—N1—C7—C1	178.2 (2)	C1—C2—C3—C4	−1.5 (5)
N2—N1—C7—C8	−1.8 (4)	C2—C3—C4—C5	−0.3 (5)
N1—N2—C9—O2	−5.8 (4)	C3—C4—C5—Cl1	−178.5 (3)
N1—N2—C9—C10	175.2 (2)	C3—C4—C5—C6	2.0 (5)
C14—N3—C13—C12	0.8 (6)	Cl1—C5—C6—C1	178.6 (2)
C13—N3—C14—C10	−1.4 (6)	C4—C5—C6—C1	−1.9 (4)
C6—C1—C2—O1	−178.0 (3)	O2—C9—C10—C11	126.3 (3)
C6—C1—C2—C3	1.6 (4)	O2—C9—C10—C14	−50.7 (4)
C7—C1—C2—O1	2.4 (4)	N2—C9—C10—C11	−54.6 (4)
C7—C1—C2—C3	−178.0 (3)	N2—C9—C10—C14	128.4 (3)
C2—C1—C6—C5	0.1 (4)	C9—C10—C11—C12	−175.7 (3)
C7—C1—C6—C5	179.7 (3)	C14—C10—C11—C12	1.3 (5)
C2—C1—C7—N1	6.1 (4)	C9—C10—C14—N3	177.6 (3)
C2—C1—C7—C8	−173.9 (3)	C11—C10—C14—N3	0.4 (5)
C6—C1—C7—N1	−173.5 (2)	C10—C11—C12—C13	−1.9 (5)
C6—C1—C7—C8	6.6 (4)	C11—C12—C13—N3	0.9 (6)

### Hydrogen-bond geometry (Å, °)

**Table:**

Cg2 is the centroid of the C1–C6 phenyl ring.

**Table:**

*D*—H···*A*	*D*—H	H···*A*	*D*···*A*	*D*—H···*A*
O1—H1···N1	0.82	1.84	2.555 (3)	144
N2—H2···O3^i^	0.86	2.06	2.898 (4)	166
O3—H3A···O2	0.89 (5)	1.88 (5)	2.760 (4)	171 (3)
O3—H3B···N3^ii^	0.91 (4)	2.01 (4)	2.885 (4)	161 (4)
C8—H8A···Cg2^iii^	0.96	2.99	3.763 (4)	139

Symmetry codes: (i) *x*−1, *y*, *z*; (ii) −*x*+1, −*y*, −*z*+1; (iii) −*x*, −*y*+1, −*z*.

## References

[bb1] Bernstein, J., Davis, R. E., Shimoni, L. & Chang, N.-L. (1995). *Angew. Chem. Int. Ed. Engl.* **34**, 1555–1573.

[bb2] Bruker (2005). *SADABS* Bruker AXS Inc., Madison, Wisconsin, USA.

[bb3] Bruker (2009). *APEX2* and *SAINT* Bruker AXS Inc., Madison, Wisconsin, USA.

[bb4] Farrugia, L. J. (1997). *J. Appl. Cryst.* **30**, 565.

[bb5] Farrugia, L. J. (1999). *J. Appl. Cryst.* **32**, 837–838.

[bb6] Shafiq, Z., Yaqub, M., Tahir, M. N., Hussain, A. & Iqbal, M. S. (2009*a*). *Acta Cryst.* E**65**, o2496. 10.1107/S160053680903709X PMC2970269 21577946

[bb7] Shafiq, Z., Yaqub, M., Tahir, M. N., Hussain, A. & Iqbal, M. S. (2009*b*). *Acta Cryst.* E**65**, o2899. 10.1107/S1600536809044134 PMC2971391 21578481

[bb8] Sheldrick, G. M. (2008). *Acta Cryst.* A**64**, 112–122. 10.1107/S0108767307043930 18156677

[bb9] Spek, A. L. (2009). *Acta Cryst.* D**65**, 148–155. 10.1107/S090744490804362X PMC2631630 19171970

